# Predictors of recurrence after a first hepatectomy for colorectal cancer liver metastases: a retrospective analysis

**DOI:** 10.1186/1477-7819-12-391

**Published:** 2014-12-20

**Authors:** Luis Cesar Bredt, Alex Francovig Rachid

**Affiliations:** Department of Abdominal Surgery, Hepatobiliary Section, Cancer Hospital-UOPECCAN, Cascavel, PR 85812-270 Brazil

**Keywords:** Liver metastases, colorectal cancer, hepatectomy

## Abstract

**Background:**

Surgical resection is considered the standard therapy in the treatment of liver metastases from colorectal cancer (CRCLM); however, most patients experience tumor recurrence after curative hepatic resection. The objective was to determine potential prognostic factors for tumor recurrence after an initial hepatectomy for CRCLM.

**Methods:**

A study population of 101 patients who had undergone a first curative hepatectomy for CRCLM was retrospectively analyzed. Selected biological tumor markers, and clinical and pathological features were then tested by Cox regression.

**Results:**

Synchronous liver metastases occurred in 38 patients (37.6%) and 63 patients (62.3%) presented with metachronous liver metastases. In a median follow-up time of 68 months, recurrence was observed in 64 patients (63.3%). The 5-year cumulative risk of recurrence was 56.7%. The median survival after recurrence was 24.5 months (range 1 to 41 months) and 5-year cumulative overall survival was 31.8%. Of all variables tested by Cox regression, intra- and extrahepatic resectable disease, CEA levels ≥50 ng/mL and bilobar liver disease remained significant as predictors of recurrence in the multivariate analysis.

**Conclusions:**

Independent risk factors for recurrence after an initial hepatectomy for CRCLM, such as intra- and extrahepatic resectable disease, CEA levels ≥50 ng/mL and bilobar liver disease, can eventually help in making decisions in this very complex scenario.

## Background

Despite recent improvements in the diagnosis and management of colorectal cancer, many cases are still diagnosed in advanced stages or relapse after curative treatment, with liver, lung or peritoneal metastases. For liver metastases, in many situations a resection is recommended, since it is currently the most effective therapy alone [1–12]. However, 50% to 75% of patients experience tumor recurrence after the first liver resection, and for only 20% to 30% of patients is the initial hepatectomy a curative treatment [4–6, 13]. For a post-hepatectomy recurrence, the treatment strategy may be very complex, and invariably includes systemic treatment possibly combined with surgical resection [14].

The identification of potential preoperative predictors of future recurrence in a candidate for liver resection for colorectal cancer liver metastases (CRCLM) is of great importance, because it may preclude or strongly indicate a liver resection, possibly combined with resection of other organs. The same is true for eventual intra-operative or pathological predictors, as long as we do not neglect unexpected resectable extrahepatic disease, unexpected macrovascular invasion, eventual resections within the R0 limit with narrow macroscopic margins, and unexpected bleeding or adherences that may interfere with the surgical planning. The aim of this study is to investigate potential clinical and pathological predictors of tumor recurrence in resected CRCLM.

## Methods

A retrospective analysis of an electronic database of prospective patients undergoing hepatectomy at the hepatobiliary section of an oncology referral center (a cancer hospital) from March 2006 until March 2010 was carried out. Only patients undergoing curative hepatectomy for CRCLM were included; patients with unresectable disease at initial surgical exploration were excluded. Before surgery, patients signed a consent form, which contained appropriate information about the surgical procedure and their inclusion in the database, which was approved by the ethics committee on human research of the Cancer Hospital – UOPECCAN, in accordance with the Declaration of Helsinki.

Epidemiological data and laboratory test data, including tumor markers, tumor characteristics, treatment, and recurrence and survival data, were analyzed to determine prognostic factors of relapse after an initial hepatectomy for CRCLM; re-hepatectomies were not included. Regarding the timing of liver metastases, synchronous CRCLM was defined as the simultaneous presentation of liver metastases and primary tumor, and as metachronous if detected after 6 months of primary tumor diagnosis.

### Preoperative imaging and tumor resectability definition

CRCLM were defined as resectable if two criteria were fulfilled: (1) oncological anticipation that the disease could be completely resected without any residual hepatic or extrahepatic disease and (2) it was possible to maintain an adequate volume of the remnant liver with preserved vascular inflow, outflow and biliary drainage. In general, at least 25% of the total liver was considered the minimum safe volume left after liver resection for patients with normal liver parenchyma, with or without preoperative portal ligature or embolization.

The diagnostic capabilities available in the preoperative staging before hepatic resection included three-dimensional computed tomography (CT) scanning, CT angiography, magnetic resonance imaging (MRI), CT volumetry and positron emission tomography (PET) combining fludeoxyglucose-PET and helical CT.

### Hepatectomy and pathological analysis

During the study, all hepatic resections were performed with curative intent by a single oncological hepatobiliary surgeon. The lymphadenectomy of the hepatic hilum was not routinely performed, being reserved for cases of suspected regional lymphatic tumor involvement. Celiac trunk lymph nodes that were positive for malignancy in an intraoperative frozen section, characterized the disease as unresectable, precluding a hepatectomy. In general, anatomical hepatectomies were performed, with the non-anatomical resections reserved for specific situations, such as two-stage resections. The transection of the liver parenchyma was performed with an ultrasonic cleaner, harmonic scalpel or kelly clasia, and the smaller vessels were ligated or coagulated by diathermy. During the procedure, the surgical margin was carefully confirmed by intraoperative ultrasonography to obtain free surgical margins. Surgical mortality was defined as death occurring within the first 30 days after the hepatectomy.

The resected specimens were studied macroscopically and microscopically to determine tumor characteristics, including size, number of tumors, morphology, macrovascular invasion according to Kondo *et al*. [15], and extent of tumor resection margins. For microscopic analysis, the samples were fixed in 10% formaldehyde and cut into sections of 5 mm and after cuts of 5 μm, and stained with H & E. Two pathologists were responsible for histological confirmation. In this study, the surgical margins were defined as the nearest distance in millimeters between the cut liver surface and the tumor.

### Chemotherapy

The decision to begin systemic treatment in the pre- or post-hepatectomy period was made in multidisciplinary meetings, and eligibility included performance status of 0 to 2 according to the Eastern Cooperative Oncology Group (ECOG) scale, the absence of systemic severe uncompensated disease, the absence of active infection, adequate hematologic parameters (white blood cell (WBC) > 4.0 × 103/L, platelets > 100 × 109/L), serum creatinine ≤1.2 mg/dL or creatinine clearance calculated by Cockcroft ≥50 mL/min, total bilirubin <5.0 mg/dL, and alanine aminotransferase and aspartate aminotransferase <100 IU/L. Regimens consisted of 5-fluorouracil (5-Fu) alone, 5-FU/leucovorin (LV), capecitabine, FOLFOX (infusional 5-FU/LV + oxaliplatin), FOLFIRI (infusional 5-FU/LV + irinotecan) and bevacizumab.

### Follow-up

Patients were followed by clinical examination, chest radiography, ultrasonography, three-dimensional CT scanning and serum carcinoembryonic antigen (CEA) every 4 to 6 months. In specific cases, MRI was used to complement CT in the diagnosis of new liver lesions, while PET or gallium scintigraphy was used for the diagnosis of systemic recurrence. Recurrences after hepatectomy for CRCLM were treated with surgical resection when resectable, systemic therapy alone or combined treatments.

### Statistical analysis

Baseline characteristics of the patients and tumor pathological features are expressed as absolute values, mean ± standard deviation and median (range) when appropriate. The length of follow-up and survival are expressed as median and ranges. Kaplan–Meier estimates of overall survival were calculated. To assess the effect of covariates on tumor recurrence, a Cox regression model was used to estimate hazard ratios (HRs) for risk factors (categorical and continuous variables). Recurrence was included as a time-dependent covariate, and significant variables in the univariate model were selected for the multivariate analysis. Cox model results are shown either as HR estimates, together with corresponding 95% confidence intervals (CIs), or as Wald’s test *P* values. According to the sample-size calculation, there was a sufficient number of events (recurrence) per variable for the multivariate analysis [16]. A value of *P =* 0.05 was considered significant. The statistical calculations were done with the SPSS for Windows 16.0 package.

## Results

### Clinical features

Of the total 101 patients, 64 were men (63.3%) and 37 were women (36.6%). The mean age at initial hepatectomy was 56.2 ± 9 years (range 36 to 79 years). The mean observation time was 68 months (range 38 to 96 months). Among the 101 patients who underwent hepatectomy for CRCLM, 38 patients (37.6%) had synchronous liver metastases with a primary tumor and in 63 patients (62.3%) there were metachronous metastases. The CEA levels before the hepatectomy were <50 ng/mL in 24 patients (23.7%) and ≥50 ng/mL in 77 patients (76.2%). Twelve patients (11.8%) received a hepatic resection simultaneously as a primary colorectal tumor resection, and 89 patients (88.1%) underwent two resection procedures. Five patients (4.9%) had resectable lung metastasis associated with hepatic metastases. Of these, two patients (1.9%) underwent simultaneous liver and lung resections. Sixty-six patients (65.3%) received pre- and post-hepatectomy systemic therapy, 12 patients (11.9%) underwent only a before hepatectomy, and seven patients (6.9%) underwent only an after hepatectomy.

### Operative data

Regarding the type of liver resection, 80 patients underwent anatomical resections (79.2%), 14 patients (13.8%) underwent non-anatomical + anatomical resections (two-stage hepatectomy), and seven patients (6.9%) underwent non-anatomical resections. Radiofrequency ablation was used during the liver resection in eight cases (7.9%). The extent of hepatic resection in 35 patients (34.6%) was less than a lobectomy and at least a lobectomy in 66 patients (65.3%). A ligature or preoperative portal embolization was performed in 24 cases (23.7%).

The surgical margins on the liver were ≥5 mm in 87 patients (86.1%), and in 14 patients (13.8%) they were <5 mm. During the hepatic resection of the parenchyma, tumors were exposed after the surface of the liver was cut in six patients (5.9%), but were not exposed for 95 patients (94.05%). The perioperative mortality in this study was 2.9%.

### Anatomopathological characteristics of liver metastases

The average size of the largest metastasis was 3.5 ± 1.3 cm. Of the 101 patients, 35 (34.6%) underwent a resection of an isolated metastasis, 36 patients (35.6%) underwent resection of two or three tumors, and in 30 patients (29.7%), four or more tumors were resected. In 79 patients (78.2%), the tumor was unilobar, in 59 cases (58.4%) there were right lobe metastases and in 20 cases (19.8%) there were left lobe metastases. Twenty-two patients (21.7%) underwent bilobar resections.

Macrovascular invasion and portal and/or hepatic vein invasion was observed in 27 patients (26.7%). A lymphadenectomy of the hepatic hilum up to the retropancreatic lymph nodes was performed when there was a clinical suspicion of regional nodal involvement at the time of hepatectomy. Lymph node metastasis was found by histopathological study for 19 patients (18.8%). A total of 24 patients (23.7%) had concomitant intra- and extrahepatic disease (lung and/or regional lymph node metastases).

### Overall survival and recurrence

**Figure 1:**
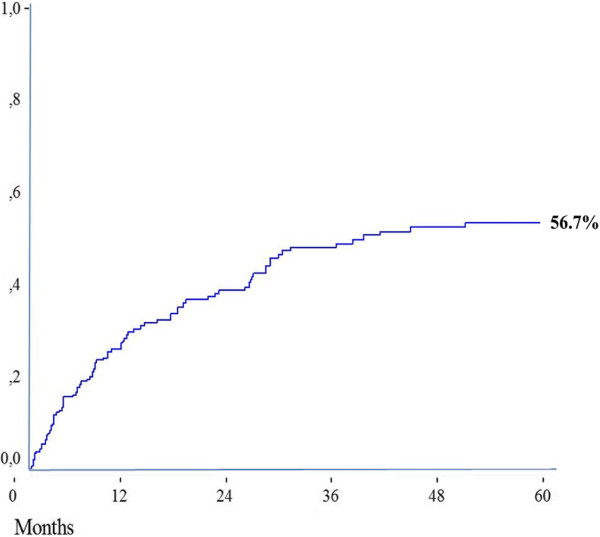
**The 5-year risk of tumoral recurrence (** ***n*** **= 101).**

**Figure 2:**
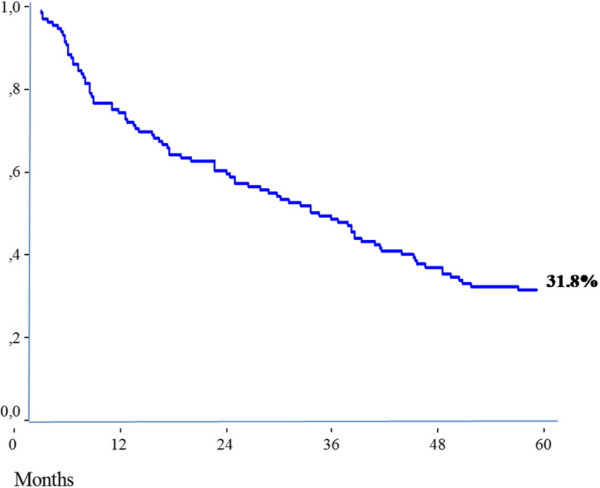
**Five-year cumulative overall survival (** ***n*** **= 101).**

Recurrence was observed for 64 patients (63.3%) during the study follow-up, and the 5-year cumulative risk of recurrence was 56.7% (Figure 1). The median survival after recurrence was 24.5 months (range 1 to 41 months) and the 5-year cumulative overall survival was 31.8%. The most common site of recurrence was the liver, which occurred for 32 patients (31.6%). There was recurrence at extrahepatic sites for only 19 patients (18.8%) and at the liver and extrahepatic sites for nine patients (8.9%) (Table 1, Figure 2).

**Table 1:** **Patterns of recurrence following hepatic resection of CRCLM (** ***n*** **= 101)**

Site of recurrence	Recurrences (%)
Overall	64 (63.3%)
Liver only	32 (31.6%)
Liver and extrahepatic	9 (8.9%)
Liver and lungs	5 (4.9%)
Liver and local	2 (1.8%)
Liver and brain	1 (0.9%)
Liver and bone	1 (0.9%)
Extrahepatic only	23 (22.7%)
Lungs	12 (11.8%)
Peritoneal cavity	4 (3.6%)
Retroperitoneal lymph nodes	2 (1.8%)
Adrenal	2 (1.8%)
Brain	1 (0.9%)
Bone	1 (0.9%)

The prognostic factors analyzed are shown in Table 2. During transection of the liver parenchyma, when tumors were exposed on the liver surface there was no association with higher recurrence rates (*P* = 0.2834). There were no differences in relapse rates when simultaneous resections of the primary tumor and liver were performed (*P* = 0.5338). Perioperative systemic therapy, whether before and/or after hepatectomy, was not associated with intra- or extrahepatic recurrence (*P* = 0.7510). Regarding the method of hepatectomy (anatomical and/or non-anatomical) and extent of liver resection (less than or at least lobectomy), there were no differences in recurrence rates between these groups (*P* = 0.1838 and 0.0967, respectively).

**Table 2:** **Cox regression of variables associated with tumor recurrence after first hepatectomy for CRCLM**

Variable	Hazard ratio (95% CI)	***P***
**Univariate**		
Sex	1.03 (0.34-3.10)	0.7418
Lobectomy	2.99 (0.93-9.60)	0.0967
Portal embolization or ligature	0.46 (0.15-1.38)	0.1672
Simultaneous resection (liver and primary)	1.37 (0.38-4.93)	0.5338
Anatomical versus non-anatomical resection	2.06 (0.83-9.05)	0.1838
Two-stage hepatectomy	2.32 (0.66-7.05)	0.278
Tumor exposed on the liver surface	4.19 (1.31-13.39)	0.2834
Perioperative systemic therapy	0.82 (0.59-2.64)	0.7510
≥4 liver metastasis	2.97 (1.73-8.25)	0.0831
Macroscopic vascular invasion	3.06 (0.85-11.05)	0.0838
Synchronous liver metastases	4.33 (0.28-9.93)	0.0433^a^
CEA before hepatectomy ≥50 ng/mL	5.51 (1.47-13.83)	0.0024^a^
Bilobar disease	5.86 (1.95-17.58)	0.0016^a^
Surgical margins <5 mm	6.32 (1.90-21.01)	0.0026^a^
Concomitant resectable intra- and extrahepatic disease	7.03 (2.27-21.80)	0.0007^a^
**Multivariate**		
Concomitant resectable intra- and extrahepatic disease	4.81 (1.61-14.37)	0.0042^a^
Bilobar disease	4.94 (1.65-14.79)	0.0049^a^
CEA before hepatectomy ≥50 ng/mL	4.13 (0.41-12.08)	0.0192^a^
Surgical margins <5 mm	1.82 (0.69-4.64)	0.7510
Synchronous liver metastases	1.23 (0.38-3.93)	0.8831

CEA, carcinoembryonic antigen.

^a^ *P* < 0.05.

In summary, the univariate analysis selected as predictors of recurrence: synchronous liver metastases (0.0433), CEA before hepatectomy ≥50 ng/mL (*P* = 0.0024), hepatic resection margins <5 mm (*P* = 0.0026), intra- and extrahepatic metastases (*P* = 0.0007), and bilobar disease (*P* = 0.0016). In the multivariate analysis, intra- and extrahepatic metastases (*P* = 0.0042), bilobar disease (*P* = 0.0049) and CEA before hepatectomy ≥50 ng/mL (*P* = 0.0192) remained significant predictors.

## Discussion

Untreated CRCLM have a poor prognosis, with median survival ranging from 6 to 12 months. Recently, increased survival after a liver resection has been found in numerous uncontrolled studies [7, 8, 13]. These studies showed 5-year survival ranging from 20% to 58%, and median survival from 24 to 46 months [4, 6–8, 10–13, 17, 18]. However, several studies have also found that patients have high recurrence rates, with intrahepatic recurrence being the most common, occurring for approximately 50% of cases [4, 6, 9, 19, 20]. An occult metastasis of the primary tumor and residual lesions have been considered as the two main pathways in which relapse occurs after the initial hepatectomy [21, 22]. Therefore, treatment strategies, including liver resection, must be considered according to the mechanisms of relapse.

Liver surgery is usually considered if the following conditions are met: (1) curative resection of the primary tumor is possible, (2) there are only liver metastases, and (3) clinically, the patient can endure a hepatectomy [23]. However, there is still a lack evidence for the ideal timing, extent of hepatectomy, resection of extrahepatic metastases and the best perioperative chemotherapy combination. The main goal of this study was to evaluate the significant prognostic factors related to recurrence in patients undergoing a first hepatectomy for CRCLM, aiming at a better understanding of this complex scenario, and mainly to help surgeons select patients to undergo liver resection with reasonable recurrence rates.

The hematogenous dissemination of colorectal cancer is significantly associated with the size of metastatic liver tumors according to the metastasis cascade theory [24], although other studies point to sporadic deviations. According to this theory, the sequence of metastatic CRCLM follows a relatively predictable process of involvement, starting from the liver, then going to the lung and advancing to other sites. However, this study did not conclude that the size or number of CRCLM are significant risk factors for recurrence.

It was found in the univariate analysis of this study that a surgical margin <5 mm was a risk factor for recurrence but was not a predictor according to the multivariate analysis. The role of surgical margin as a prognostic factor is still a matter of controversy [25–29]. In a large series, it was shown that a margin >1 cm is an independent predictor of survival [28]. Recently, it was found that a histological surgical margin ≤5 mm was associated with a lower disease-free interval and worse survival rates [29]. In contrast, another study showed that positive margins were not associated with an increased risk of relapse [25].

Various devices for coagulation of the liver parenchyma transection can be potentially detrimental to a surgical margin evaluation [24, 29, 30], but in practical terms, hepatectomy should not be contraindicated whatever the margin thickness, since no other treatment modality alone is better than resection, even with narrow margins that range from 0 to 1 cm [28]. In the present study, as already mentioned, the surgical margin was a prognostic factor according to the univariate analysis, but not according to the multivariate analysis.

Although more studies are needed to clarify the clinical significance of macroscopic vascular invasion, this reflects the degree of aggressiveness of the tumor and the potential for intrahepatic micrometastases [29, 31], thus it can be used to estimate the malignant potential. Therefore, the optimal surgery should include strategies to deal with macroscopic vein invasion, such as anatomical liver resection including a Glisson sheath [31].

Another important point is the possibility of performing simultaneous resections of the liver and the primary site or of performing resections separated by an interval. No differences between these two approaches were found in this study, suggesting that synchronous metastases at surgery can be resected before the hepatic tumor becomes inoperable, and without the necessity of compulsory neoadjuvant treatment.

Controversy remains whether the actual consensus for perioperative chemotherapy for resectable or potentially resectable liver metastases is significantly associated with disease-free survival or prognosis [32]. The effects of the combination of surgery and systemic therapy were not the object of this study, though apparently in the subgroup of patients with synchronous CRCLM, adjuvant systemic treatment seems essential. Chemotherapy in this study relied primarily on 5-FU alone, 5-FU/LV, capecitabine, FOLFOX, FOLFIRI and bevacizumab. Recently developed molecular target agents, such as bevacizumab, have been reported in the literature as strategies that can improve the prognosis of recurrent and unresectable colorectal cancer [33–35].

A systematic review by Park *et al*. [36] concluded that systemic adjuvant chemotherapy gave a significant improvement in disease-free survival, and indicated that choice for patients with liver metastases from colorectal cancer after resection in those with a high likelihood of recurrence. The most suitable systemic chemotherapy regimen is 5-FU/LV, but many oncologists are using systemic FOLFOX perioperatively due to the observed longer disease-free survival. According to Macedo *et al*. [37], adjuvant chemotherapy with 5-FU is the most used regime in clinical practice, with a trend for improvement of disease-free survival, but with no difference in overall survival or median disease-progression-free survival. A retrospective analysis by Boame *et al*. [38] of 168 patients of Ottawa Cancer Hospital compared the use of adjuvant, neoadjuvant and perioperative chemotherapy for patients with liver metastases from colorectal cancer, and the conclusion was that regardless of when they received chemotherapy, these patients had improved overall survival and disease-free survival.

Currently, the recommended approach for the neoadjuvant scenario is 2 to 3 months of FOLFOX, but there are potential negative effects of chemotherapy, such as the development of extrahepatic lesions and the appearance of postoperative sequelae [37]. The review by Park *et al*. [36] also shows that when the conversion of an unresectable disease is needed, the rates are 15%, 30% and >50% with the use of FOLFIRI or FOLFOX, regional hepatic artery infusion of floxuridine, and hepatic artery infusion with FOLFOX, respectively. In the neoadjuvant setting, response rates with bevacizumab and FOLFOX of 60% and 70%, respectively, have been reported. The hepatic artery infusion technique was also used by Osawa *et al*. [39], with complete response in a patient with CRCLM after 5-FU infusion for 26 weeks.

Although substantial conclusions for this subject were not demonstrated in this study, mainly because of the study design, the use of these new agents may have a positive impact on the control of relapses by the probable extermination of micrometastases.

Another point of interest is the challenging bilobar liver metastases. Although some patients in this study did benefit from aggressive neoadjuvant chemotherapy until the lesions reduced in size, and then subsequently underwent a hepatectomy, bilobar disease was an independent prognostic factor for recurrence in this study. The modalities of increasing resectability in this setting include radiofrequency ablation [40, 41], percutaneous transportal embolization [42] and hepatectomy in two stages [43].

Ribeiro *et al*. [44] revealed that CEA levels >200 ng/mL are associated with poor results. Other studies agree with this because they cannot demonstrate a clear disadvantage for survival when there is a high preoperative CEA level. Torrez [45] points out that favorable survival at 5 years was observed for patients with a serum CEA level less than 50 ng/mL, 34.4% versus 18.8% (*P* ≤ 0.001). In our study, patients with serum CEA levels above 50 ng/mL had a poorer prognosis.

The comparative prognosis for CRCLM, either synchronous or metachronous, still remains unclear in the literature. A systematic review by Tan and Ooi [46] showed that 14.5% to 24% of patients had synchronous metastases, and 8.1% to 20% of patients had metachronous metastases. The authors found that survival rates at 5 and 10 years were 16% to 44% and 20% to 30.9%, respectively. This study has shown there is a difference in survival, which favors metachronous lesions, although statistically significant differences were not always found. As the review of Tam *et al*., this study also found a trend for more favorable prognosis with metachronous lesions, but this was not confirmed by the multivariate analysis. Tan and Ooi [46] also reported that despite not finding differences in survival, there was a shortening of the disease-free interval after 5 years (18%) for synchronous metastases. Ghirimghelli *et al*. [47] conducted a population-based study of 932 patients with metastatic colon cancer. The 5-year survival for metachronous metastases was 17.6% while for synchronous metastases it was 7.2%. Other studies affirm that there are no statistically significant differences for prognosis and overall survival between synchronous and metachronous metastases [48–50].

The criteria for resection of liver metastases have been recently improved; previously the surgical therapy was grounded in how much of the liver was removed. Nowadays, what remains after resection is what matters. Characteristics as the number of liver metastases, the size of the tumors and surgical margins of 1 cm are not static criteria of unresectability anymore. Currently, lesions must be removed with negative margins leaving an appropriate remnant liver volume. Moreover, patients with intra- and extrahepatic metastases are now considered as potential candidates for resection [13]. Angelsen *et al*. [51] reported the 5-year survival for patients undergoing a R0 resection as 42.5%, and 16.1% for R1. Park *et al*. [36] point out that in the 1980s, only 10% of patients were candidates for resection, and only those with solitary lesions or with less than four unilobar lesions were eligible. Surgery is now considered for patients with more extended disease, and excellent long-term results can be achieved. Thus, it is estimated that over 50% of patients are potential candidates for hepatectomy. Concomitant extrahepatic disease has traditionally been related to a formal contraindication for liver resection; however, studies have shown an increase in overall survival of patients with extrahepatic disease who underwent hepatectomy and extrahepatic disease resection with curative intent [44]. For example, surgery may be beneficial in cases of hepatic pedicle lymph node involvement (38% survival at 3 years) [52, 53]. For lung metastases, resection can be performed safely with low mortality (0.0% to 2.5%) and overall survival of 24% to 64% at 5 years [44, 54, 55]. Our results revealed higher recurrence rates for concomitant resectable extrahepatic disease.

## Conclusions

We can conclude that the currently available treatments for CRCLM are efficient, but not completely satisfactory, and the independent risk factors for tumor recurrence identified in this study can eventually help in making decisions for this very complex scenario. Because of the heterogeneity of these patients and variable disease biology, more studies are needed to clarify this issue.

## Abbreviations

5-Fu: 5-fluorouracil
CEA: carcinoembryonic antigen
CI: confidence interval
CRCLM: colorectal cancer liver metastases
CT: computed tomography
FOLFIRI: infusional 5-FU/LV + irinotecan
FOLFOX: infusional 5-FU/LV + oxaliplatin
H & E: hematoxylin and eosin
HR: hazard ratio
LV: leucovorin
MRI: magnetic resonance imaging
PET: positron emission tomography.
